# Localization, Proteolytic Processing, and Binding Partners of Versican Isoforms in Vascular Lesions of Pulmonary Arterial Hypertension

**DOI:** 10.1369/00221554251331271

**Published:** 2025-04-11

**Authors:** Christian Westöö, Ayse Ceren Mutgan, Oscar van der Have, Timothy J. Mead, Salaheldin Ahmed, Elna Lampei, Christopher D. Koch, Christian Norvik, Anders Aspberg, Martin Bech, Niccolò Peruzzi, Hans Brunnström, Grazyna Kwapiszewska, Göran Rådegran, Suneel S. Apte, Karin Tran-Lundmark

**Affiliations:** Department of Experimental Medical Science, Lund University, Lund, Sweden; Wallenberg Centre for Molecular Medicine, Lund University, Lund, Sweden; Department of Experimental Medical Science, Lund University, Lund, Sweden; Wallenberg Centre for Molecular Medicine, Lund University, Lund, Sweden; Lung Research Cluster, Otto Loewi Research Center, Medical University of Graz, Graz, Austria; Department of Experimental Medical Science, Lund University, Lund, Sweden; Wallenberg Centre for Molecular Medicine, Lund University, Lund, Sweden; Children’s Heart Centre, Skåne University Hospital, Lund, Sweden; Department of Biomedical Engineering, Cleveland Clinic Lerner Research Institute, Cleveland, OH; Department of Pediatrics, School of Medicine, Case Western Reserve University, Cleveland, OH; University Hospitals Rainbow Babies & Children’s Hospital, Cleveland, OH; Department of Clinical Sciences Lund, Cardiology, Lund University, Lund, Sweden; The Hemodynamic Lab, The Section for Heart Failure and Valvular Disease, VO. Heart and Lung Medicine, Department of Clinical Science Lund, Cardiology, Skåne University Hospital, Lund, Sweden; Department of Education and Research, Helsingborg Hospital, Helsingborg, Sweden; Department of Experimental Medical Science, Lund University, Lund, Sweden; Wallenberg Centre for Molecular Medicine, Lund University, Lund, Sweden; Children’s Heart Centre, Skåne University Hospital, Lund, Sweden; Department of Biomedical Engineering, Cleveland Clinic Lerner Research Institute, Cleveland, OH; Department of Pathology, Sanford School of Medicine, University of South Dakota, Sioux Falls, SD; Sanford Laboratories, Sanford Health, Sioux Falls, SD; Department of Experimental Medical Science, Lund University, Lund, Sweden; Wallenberg Centre for Molecular Medicine, Lund University, Lund, Sweden; Department of Clinical Sciences Lund, Rheumatology and Molecular Skeletal Biology, Lund University, Lund, Sweden; Medical Radiation Physics, Department of Clinical Sciences, Lund University, Lund, Sweden; Department of Experimental Medical Science, Lund University, Lund, Sweden; Wallenberg Centre for Molecular Medicine, Lund University, Lund, Sweden; Medical Radiation Physics, Department of Clinical Sciences, Lund University, Lund, Sweden; Department of Clinical Sciences Lund, Division of Pathology, Lund University, Lund, Sweden; Department of Genetics and Pathology, Division of Laboratory Medicine, Region Skåne, Lund, Sweden; Lung Research Cluster, Otto Loewi Research Center, Medical University of Graz, Graz, Austria; Institute for Lung Health, German Center for Lung Research (DZL), Cardio Pulmonary Institute, Giessen, Germany; Department of Clinical Sciences Lund, Cardiology, Lund University, Lund, Sweden; The Hemodynamic Lab, The Section for Heart Failure and Valvular Disease, VO. Heart and Lung Medicine, Department of Clinical Science Lund, Cardiology, Skåne University Hospital, Lund, Sweden; Department of Biomedical Engineering, Cleveland Clinic Lerner Research Institute, Cleveland, OH; Department of Experimental Medical Science, Lund University, Lund, Sweden; Wallenberg Centre for Molecular Medicine, Lund University, Lund, Sweden; Children’s Heart Centre, Skåne University Hospital, Lund, Sweden

**Keywords:** proteolysis, pulmonary vascular disease, three-dimensional imaging, versican, versikine

## Abstract

Pulmonary arterial hypertension (PAH) is a lethal condition where expansion of the vascular extracellular matrix contributes to increased pulmonary vascular resistance. Versican, a chondroitin sulfate proteoglycan, is known to accumulate in vascular lesions of PAH and hyaluronan and tenascin-C, binding partners of versican, are elevated in PAH. The specific distribution and localization of versican isoforms, their cleavage products, and binding partners in vascular lesions of PAH had not been studied previously. Versican has five distinct isoforms, V0–V4, identified by the arrangement of its chondroitin-sulfate attachment regions, GAGα and GAGβ. Here, tissue from idiopathic PAH was imaged with synchrotron-based phase-contrast micro-CT and analyzed by histology, immunohistochemistry, and in situ hybridization. Plasma concentration of versican in PAH patients and controls was measured using ELISA. GAGα- and GAGβ-containing isoforms were identified in pulmonary arteriopathy of all patients. However, immunohistochemical staining of N-terminal G1 domain (versican G1) and C-terminal G3 domain (versican G3) using specific antibodies did not consistently co-localize. Tenascin-C was occasionally found in neointima, but also in thin-walled collateral vessels. Hyaluronan accumulated in the neointima, co-localizing with both versican G3 and the neoepitope DPEAAE. DPEAAE did not co-localize with the corresponding neoepitope of the C-terminal fragment generated by cleavage, possibly indicating motility of fragments. Patient plasma had a higher concentration of versican G3-containing fragments, compared to controls. The distribution of versican isoforms, cleavage products, and binding partners demonstrated here warrants further investigation of their functional roles in PAH, versican G3 was reinforced as a potential biomarker for PAH.

Pulmonary arterial hypertension (PAH) is a condition with elevated mean pulmonary arterial pressure. Pre-capillary vasoconstriction combined with obliterative vascular remodeling reduces the luminal area of pulmonary arteries, thereby increasing pulmonary vascular resistance (PVR).^1^ Owing to the lack of curative therapy PAH will, with time, result in end-stage right-heart failure.^2^ Although available treatments reduce PVR and prolong survival, there is an urgent need for novel therapies that effectively target the underlying vascular remodeling.

Remodeling of pulmonary arteries and arterioles in PAH includes hypertrophy of the tunica media, intimal hyperplasia (neointima formation), and development of plexiform lesions, the hallmark vasculopathy of advanced PAH.^3,4^ Pulmonary arterial smooth muscle cells (PASMCs) are thought to play an important role in occlusive vascular lesions through their proliferation, migration, and extracellular matrix (ECM) production.^5^ Versican, a member of the lectican/hyalectan family of ECM proteoglycans, is produced by synthetic PASMCs and accumulates in vascular lesions of PAH.^6–9^ The versican core protein consists of an N-terminal G1 domain (versican G1) and a C-terminal G3 domain (versican G3), with specific combinations of alternatively spliced glycosaminoglycan (GAG) attachment regions (GAGα and GAGβ), generating five different isoforms (V0–V4). The four widely recognized versican isoforms, V0–V3, are shown in Fig. 1. V0, containing both GAGα and GAGβ, is expressed widely during embryonic development.^10^ V1, containing only GAGβ, is the most abundant and widely distributed versican isoform in adult tissues ^11^ and was shown to promote proliferation and inhibit apoptosis^12^ in the context of inflammation.^13^ Isoform V2, containing GAGα, on the contrary, is mostly found in the central nervous system.^14^ The expression of V3, lacking both GAG-bearing regions, is low in adult tissues^15^ and is not elevated in pathological conditions to any significant extent.^11^ Little is known about the location and function in humans of isoform V4, resulting from alternative splicing in exon 8, affecting the N-terminus of the GAGβ domain.^16^

**Figure 1.**
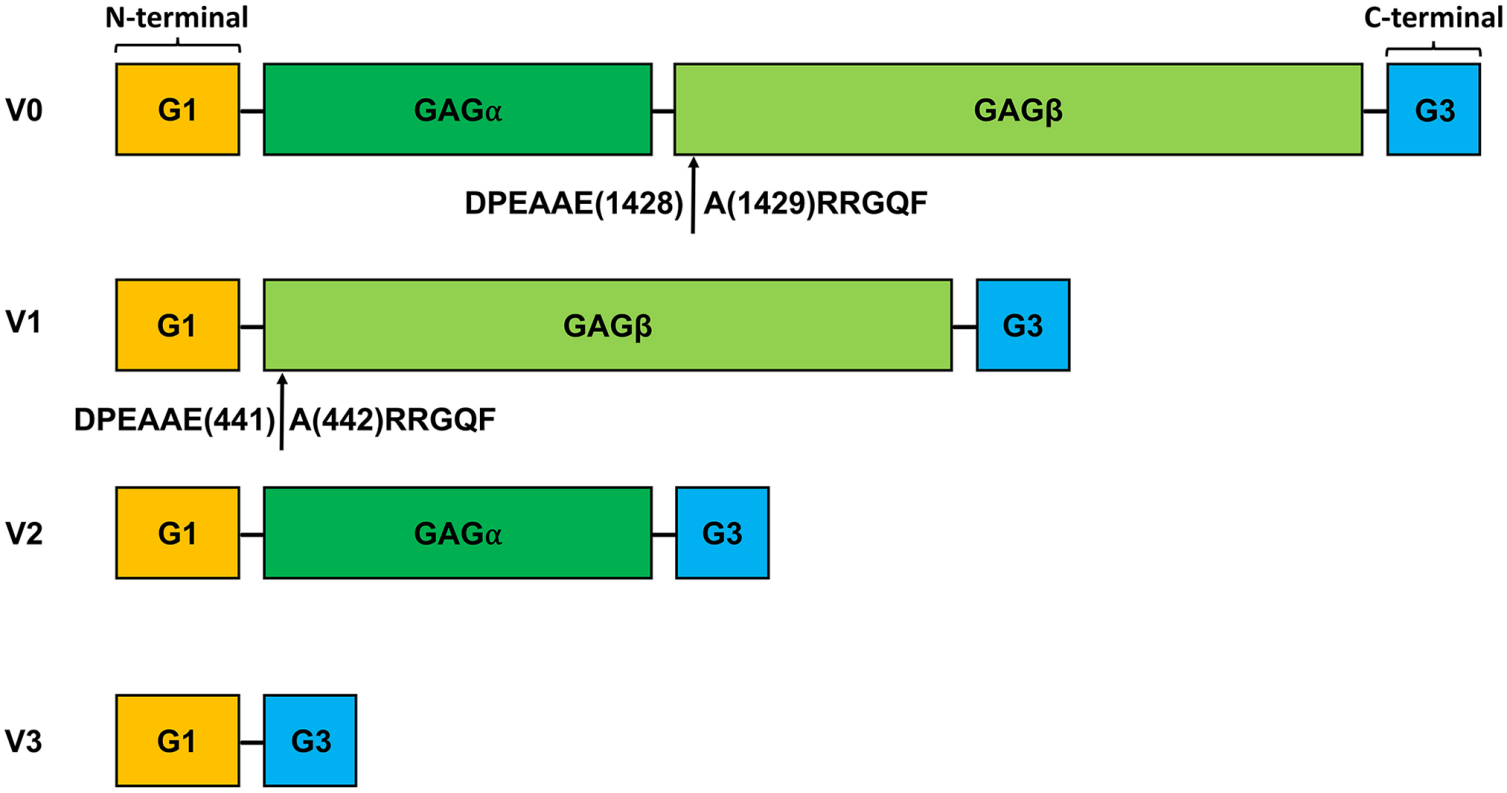
Versican and its isoforms V0–V3. Schematic illustration of versican, showing the four canonical isoforms. Versican isoforms comprise an N-terminal G1 domain (binding region for HA) and a C-terminal G3 domain (binding domain for Tenascin-C), with alternatively spliced glycosaminoglycan (GAG) attachment domains GAGα and GAGβ. Isoform V0 contains both GAGα and GAGβ, V1 only GAGβ, and V2 only GAGα. Isoform V3 lacks GAG domains. Arrows mark cleavage sites in the GAGβ domain for ADAMTS proteases that generate fragments with neoepitopes DPEAAE and ARRGQF at site Glu1428-Ala1429 in isoform V0 and Glu441-Ala442 in isoform V1.

Among the binding partners of versican, hyaluronan (HA) and tenascin-C are elevated in the circulation of PAH patients.^17-19^ Hyaluronan is a non-sulfated ECM GAG with a wide range of biological functions.^20^ It binds to versican through the N-terminal G1 domain and creates pericellular matrices that facilitate migration and proliferation of vascular smooth muscle cells.^21^ Both versican and HA accumulate in re-stenotic vascular lesions of the systemic circulation.^21^

Tenascin-C, a glycoprotein that crosslinks HA-lectican complexes, is a high-affinity ligand for the versican G3 domain.^22,23^ Although predominantly expressed during embryonic development, tenascin-C is expressed during wound healing and in vascular disease.^24,25^ Even though elevated levels of HA and tenascin-C were reported in PAH, their localization and spatial relationship to versican in PAH vascular lesions had yet to be elucidated.

Cleavage in the GAGβ domain of isoform V0 and V1 by several members of the ADAMTS (a disintegrin-like and metalloproteinase domain with thrombospondin type 1 motifs) protease family generates an amino-terminal fragment with the C-terminal neoepitope sequence DPEAAE.^26^ This fragment, when generated from isoform V1, is biologically active, and was named versikine.^27–29^ Mice expressing ADAMTS-cleavage-resistant versican exhibited significantly faster wound healing, suggesting an effect of versican accumulation or an inhibitory effect of the N-terminal fragment.^30^ ADAMTS-mediated turnover of versican and the location of its proteolytic products were not previously studied in vascular lesions of PAH.

The objective of this work was to investigate the distribution of different isoforms of versican in PAH, to explore proteolytic turnover mediated by ADAMTS proteases, and to localize binding partners of versican in PAH arteriopathy. For localization in a three-dimensional context, immunohistochemistry was aligned with synchrotron-radiation phase-contrast micro-computed tomography (SRµCT). A related objective was to test a recent finding^8^ of increased versican fragments in plasma from PAH patients in our cohorts.

## Methods

### Patient Tissue and Plasma Collection

Patient tissues and plasma were collected between 2003 and 2014. Idiopathic PAH (IPAH) was defined as PAH, according to the hemodynamic criteria at the time of diagnosis,^31^ without any other known cause of PAH. Hereditary PAH (HPAH) was defined as PAH, according to the hemodynamic criteria at the time of diagnosis, but with a known genetic abnormality associated with HPAH.

Archived paraffin-embedded pulmonary tissue cataloged as IPAH, acquired at lung transplant, was reviewed and obtained from the pathology biobank at Skåne University Hospital, Lund, Sweden (*n* = 10). Donor lungs not utilized for transplantation were used as controls (*n* = 3). Tissue sections (4 μm) were prepared by the Department of Pathology, Skåne University Hospital. Patient characteristics for the tissue cohort are summarized in Table 1.

**Table 1.** Clinical Characteristics of Included Patients.

	Tissue Cohort (*n = 10*)	Plasma Cohort I (*n = 10*)	Plasma Cohort II (*n = 10*)
Sex, M/F	1/9	3/7	1/9
Age at diagnosis, years	27 (22–44)^a^	43 (30–52)^a^	57 (38–66)^a^
Disease duration, years	2.5 (1–7.3)^a^	ND	ND
PAH subtype, IPAH/hPAH	10/0	7/3	9/1
PAH medication	10 (100%)	10 (100%)	0 (0%)
	Prior to LTX	Clinical evaluation	
Age, years	32 (24–45)^a^	41.5 (29–51)^a^	57 (38–66)^a^
BMI, kg/m^2^	23 (20–24)^a^	27 (23–30)^a^	29 (24–30)^a^
WHO functional class^b^			
I, *n* (%)	0 (0%)	1 (11%)	0 (0%)
II, *n* (%)	0 (0%)	8 (89%)	2 (20%)
III, *n* (%)	4 (40%)	0 (0%)	8 (80%)
IV, *n* (%)	6 (60%)	0 (0%)	0 (0%)
6MWT, meter	308 (205–385)^a^	433 (325–500)^a^	255 (205–355)^a^
	Hemodynamics prior to LTX	Hemodynamics at clinical evaluation	
SaO_2_, %	93 (89–96)^a^	92.5 (92–96)^a^	94 (92–96)^a^
Hb, g/L^c^	140 (131–156)^a^	ND	163 (145–166)^a^
mAP, mmHg	83 (75–93)^a^	96 (92–100)^a^	101 (96–109)^a^
mPAP, mmHg	68 (58–82)^a^	53.5 (42–72)^a^	52.5 (49–55)^a^
PVR, WU	12.5 (11.5–25.4)^a^	7 (3.0–10.0)^a^	11.5 (9.8–14.7)^a^
Supra-systemic mPAP, *n* (%)	3 (30%)	1 (10%)	0 (0%)

LTX, lung transplantation; BMI, body mass index; WHO, World Health Organization; 6MWT, non-encouraged 6-minute walk test; SaO_2_, arterial oxygen saturation; Hb, hemoglobin; mAP, mean arterial pressure; mPAP, mean pulmonary arterial pressure; PVR, pulmonary vascular resistance; WU, wood units; ND, no complete data available; IPAH, idiopathic pulmonary arterial hypertension; HPAH, hereditary pulmonary arterial hypertension.

a Median with interquartile range (IQR).

b WHO functional class data were unavailable for one patient in the plasma Cohort I.

c Hemoglobin data were unavailable for seven patients in plasma Cohort II.

Non-fasting venous blood samples were collected from IPAH and HPAH patients’ introducers during routine diagnostic right heart catheterization procedures. Samples were collected both from patients that had received PAH specific treatment (*n* = 10, PAH Cohort I) and from patients that had not received PAH specific treatment (*n* = 10, PAH Cohort II). Peripheral venous samples (*n* = 10) were collected from healthy controls and used as controls for both Cohort I and Cohort II. After blood collection in ethylenediaminetetraacetic acid (EDTA) vacutainer tubes, the samples were centrifuged at 2000 × g for 10 minutes at RT and subsequently stored at −80°C in the Lund Cardio-Pulmonary Registry (LCPR) within Region Skåne’s Biobank. Plasma cohorts I and II were actively selected to match the hemodynamic parameters of the tissue cohort. Patient characteristics for Cohort I and Cohort II are summarized in Table 1.

All specimens used for this project were de-identified. Permission to access clinical data was granted by the Skåne County Council (KVB Dnr 235-20), and the study was approved by the regional ethical review board in Lund, Sweden (Dnr 2017/597, Dnr 2019-01769, Dnr: 2010/114, 2011/368, 2015/270). This study complies with the ethical principles outlined in the declarations of Helsinki and Istanbul.

### Synchrotron-Based Phase-Contrast Micro-Computed Tomography; Image and Tissue Processing

SRµCT data acquisition and processing were performed as previously described.^32–34^ Briefly, a single 4-μm section from each IPAH specimen was stained with hematoxylin according to standard protocols, scanned, and digitalized with Aperio Scanscope CS2 (Leica Biosystems, Nussloch, Germany). Areas with vascular pathology were selected for subsequent beam alignment. Each area of interest was scanned with 80-ms single-projection exposure time and 1,801 tomographic projections whilst rotating the sample 180°. Phase information retrieval was done using the algorithm by Paganin et al.^35^ and tomographic reconstruction was performed using a Fourier-based re-gridding algorithm.^36^ ImageJ^37^ and Amira^®^ (Thermo Fisher Scientific, Waltham, MA) were used for data/image processing. Manual segmentation was performed by tracing structures (vessels, airways) of interest, marking every 5th to 20th slice, and interpolating the intermediate slices. Following imaging, tissue blocks from four patients were serially sectioned (4 μm thick), with every 10th section stained. Between 20 and 30 sections were prepared from the remaining tissue blocks. Fresh 6-μm thick sections were obtained for RNA in situ hybridization (ISH) from blocks that had not undergone serial sectioning.

### Immunohistochemistry, Immunofluorescence, and ISH

Immunohistochemical/immunofluorescent staining for versican core protein domains G1 and G3, versican neoepitopes DPEEAE and ARRGQF, GAGα and GAGβ domains, α-smooth muscle actin, von Willebrand factor, HA, and tenascin-C were performed as previously described.^7^ Appendix Information I1 and I2 and Appendix Table 1 provide details of experimental protocols and primary/secondary antibodies used, as well as the number of replicate stainings. Immunohistochemistry and immunofluorescence were repeated on three separate occasions for all IPAH tissue and donor lung controls, except for control experiments for antibody specificity. Representative images are shown. RNA ISH was performed using RNAscope (Advanced Cell Diagnostics, Newark, CA).^38^ Fresh 6-µm human lung sections were deparaffinized and hybridized to the human *VCAN* exon 7 and exon 8 probe sets (Advanced Cell Diagnostics, Cat No. 452238 and 452248) using a HybEZ^TM^ oven (Advanced Cell Diagnostics) and the RNAscope 2.5 HD Detection Reagent Kit (322360; Advanced Cell Diagnostics). Slides were then incubated in blocking solution (5% goat serum in phosphate-buffer saline [PBS] 0.01% Triton X-100) for 1 hr prior to overnight incubation with anti-α smooth muscle actin or anti-von Willebrand factor overnight at 4°C, allowing for combined ISH and immunofluorescent staining. This was followed by secondary goat anti-mouse or goat anti-rabbit antibody and incubation with DAPI. All images were captured using AxioPhot 2 Fluorescent Microscope (Zeiss, Oberkochen, Germany) with a C4742-95 camera (Hamamatsu Photonics, Hamamatsu, Japan) and an X-cite series 120Q lamp (Lumen Dynamics, Mississauga, Canada). Images were merged and processed using Openlab v 5.0.2 (Improvision^®^, Coventry, England).

### Enzyme-Linked Immunosorbent Assay

For quantitative measurement of versican in plasma, an enzyme-linked immunosorbent assay (ELISA) was used according to the manufacturer’s instructions (CSB-E11884h, Cusabio Technology, Houston, TX). Plasma specimens were retrieved from −80°C, thawed on ice, and diluted at 1:240 with sample diluent provided in the kit. An Epoch table-top microplate spectrophotometer (Agilent BioTek, Santa Clara, CA) was used for measurements at wavelength 450 and 540 nm for wavelength correction. Samples from patients and controls, as well as the standard, were analyzed in triplicates or duplicates. ELISA experiments were repeated on four separate occasions for all initial patients and controls, and an additional experiment was performed for a separate cohort of patients to extend and validate the findings. Standard curves were calculated using the online data analysis tool MyAssays^®^ (Brighton, UK). In validation experiments, the ELISA assay showed no reaction with mammalian cell expressed recombinant human versican G1 domain, and mass spectrometry of the assay kit standard confirmed it was the versican G3 protein domain (data not shown).

### Statistical Analysis

Statistical analyses were performed in GraphPad Prism version 8.1.1. Data normality distribution were assessed with histograms, normality tests (Shapiro–Wilk), Q-Q plots, and skewness. Clinical variables are described using frequencies and percentages, whereas medians and interquartile range (IQR) were used for continuous variables. Continuous data were compared using unpaired independent samples *t*-test. For ELISA experiments, versican concentrations were calculated using a four-parametric logistic curve using the online data analysis tool MyAssays^®^. A two-sided *p* value <.05 was considered significant.

## Results

### Patient and Tissue Characteristics

All patients contributing tissue or plasma fulfilled the clinical criteria for IPAH or HPAH (HPAH only for the plasma cohort) at the time of diagnosis.^31^ Clinical data for the included PAH patients contributing tissue and plasma are listed in Table 1. Clinical data on the tissue cohort was previously reported by our group.^7,33^ All acquired lung tissue showed irreversible, end-stage, pulmonary vascular remodeling of IPAH phenotype, including neointima, hypertrophy of the tunica media, and plexiform lesions.^4^ These vascular changes were not observed in the donor lung control group.

### Expression and Distribution of GAGα and GAGβ in IPAH Vasculopathy

**Figure 2.**
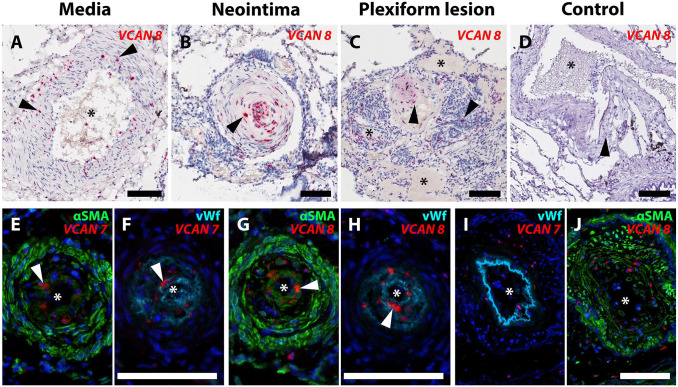
Local production of GAGα and GAGβ in IPAH vasculopathy. In situ hybridization (ISH) for *VCAN* (versican mRNA) in pulmonary arterial hypertension (PAH) lesions. Asterisks mark the vascular lumen in all images. A-C. mRNA containing *VCAN* exon 8 (GAGβ) was identified in hypertrophied tunica media (A), intimal occlusions (B), and plexiform lesions (C). D. Healthy tissue. Arrowheads indicate signal with the *VCAN exon 8* probe set (red). E-J. ISH for *VCAN* (exon 7 and exon 8) detected using fluorescence (red) combined with immunofluorescence for α-smooth muscle actin (αSMA, green) and von Willebrand factor (vWF, turquoise). Images E through H show sections of the same vascular lesion with a prominent neointima. Both αSMA- and vWF-positive cells expressed *VCAN exon 7* in E and F, and *exon 8* containing splice isoforms in G and H (white arrowheads show examples of positive ISH signal in E-H). Images I and J show a vascular lesion with prominent hypertrophy of the tunica media, where both *VCAN exon 7* (I) and *VCAN exon 8 probes* (J) were localized. Scalebars = 100 µm.

**Figure 3.**
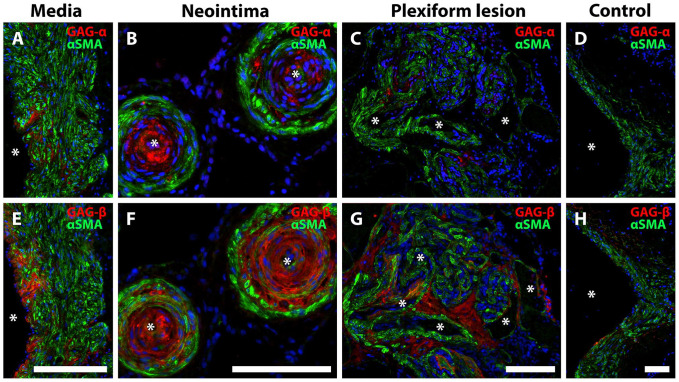
GAGα and GAGβ immunostaining in IPAH vasculopathy. Immunofluorescence for versican GAGα (A-D) and GAGβ (E-H) (red). Asterisks mark the vascular lumen in all images. αSMA is shown in green. A, E, tunica media; B, F, neointima; C, G, plexiform lesion; D, H, healthy vascular wall. Note that the area with positive staining was larger for GAGβ than GAGα, although both were localized to the tunica media (A, E), neointima (B, F), and plexiform lesions (C, G). Almost no positive signal for GAGα, and very little for GAGβ was observed in healthy (control) vascular tissue (D, H). Scalebars = 100 µm.

ISH revealed the expression of *VCAN* mRNA (Fig. 2) within neointimal lesions and hypertrophic tunica media, as well as in plexiform lesions (Fig. 2A to D). No apparent difference in pattern of expression was observed when comparing *VCAN* exon 7 (GAGα) and *VCAN* exon 8 (GAGβ) ISH. Cells producing *VCAN* were positive for smooth muscle α-actin (αSMA), but both *VCAN* exon 7 and *VCAN* exon 8 were also found within von Willebrand factor-positive endothelial cells, as well as in cells negative for both these markers (Fig. 2E to J). Immunostaining for GAGα and GAGβ revealed the presence of both domains in lesions of IPAH (Fig. 3). The staining intensity was greatest in the neointima (Fig. 3B and F) for both epitopes, with a less-intense and more-heterogeneous signal in tunica media (Fig. 3A and E). GAGβ was present in larger areas of the lesions compared with GAGα; a pattern observed across the neointima, tunica media and in plexiform lesions (Fig. 3A to C, E to G). In healthy tissue, very little GAGβ and no GAGα staining was observed (Fig. 3D and H).

### Proteolytic Turnover of Versican in IPAH

**Figure 4.**
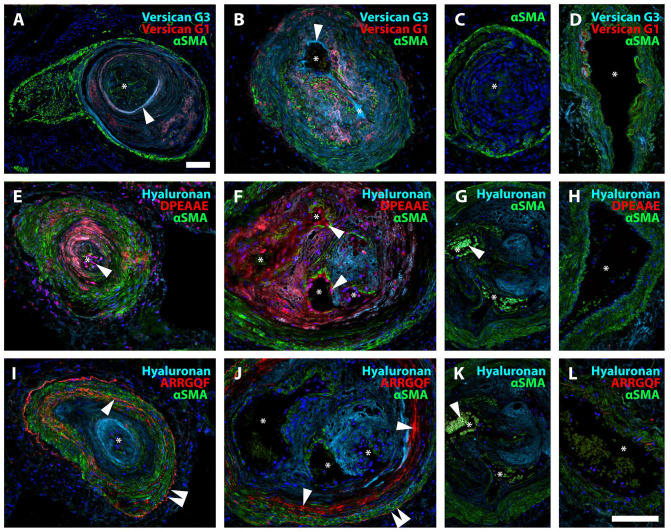
Proteolytic turnover of versican in IPAH. A, B. Neointimal lesions showing versican G3 (turquoise), versican G1 (red), and αSMA (green). Note the distinct separation of G1 and G3 staining, consistent with redistribution after versican cleavage. Staining for versican G3 appeared closer to the vascular lumen than the signal for versican G1 as demonstrated in A and B (arrowheads). C. Negative control. D. Healthy control, where very little positive staining was observed for versican G3 and G1. E, F. DPEAAE (red) was primarily localized to the neointima, colocalizing with hyaluronan (turquoise), and in the endothelium. Arrowheads indicate the DPEAAE signal in endothelial cells. G. Specificity control. Patient tissue, antibody pre-incubated with DPEAAE peptide. The white arrow indicates auto-fluorescent erythrocytes. H. Healthy control. I, J. ARRGQF (red) was consistently found in a circular pattern at the border between the neointima and tunica media (single arrowhead). A weaker signal was also often seen at the outer border of the tunica media (double arrowhead). Note the abundant signal for hyaluronan (turquoise) in the neointima. K. Specificity control. Patient tissue, antibody pre-incubated with ARRGQF peptide. The white arrow indicates auto-fluorescent erythrocytes. L. Healthy control. Asterisks mark the vascular lumen in all images. Scalebars = 100 µm; the scalebar in B is applicable for B-L.

Versican G1 (N-terminus) and G3 (C-terminus) staining did not show consistent co-localization, suggesting proteolytic cleavage and separation of the resulting fragments (Fig. 4). When distinct separation of G1 and G3 was apparent in PAH lesions, neointimal G1 appeared to localize near the tunica media and G3 closer to the vascular endothelium (Fig. 4A to D). Staining for DPEAAE neoepitope (C-terminus of the N-terminal fragment resulting from cleavage by ADAMTS proteases, Fig. 4E to H) and ARRGQF (N-terminus of the C-terminal fragment created by the same ADAMTS cleavage, Fig. 4I to L) revealed distinct patterns of distribution, further supporting the possibility of fragment redistribution. DPEAAE was mainly localized around α-smooth muscle actin-negative cells within the neointima and along the endothelial lining (Fig. 4E to H). DPEAAE frequently colocalized with HA, which was consistently abundant in the neointima. ARRGQF was almost invariably observed at the border between neointima and tunica media. HA did not colocalize with ARRGQF (Fig. 4I to L).

### Neointimal Accumulation of Versican Binding Partners in IPAH

**Figure 5.**
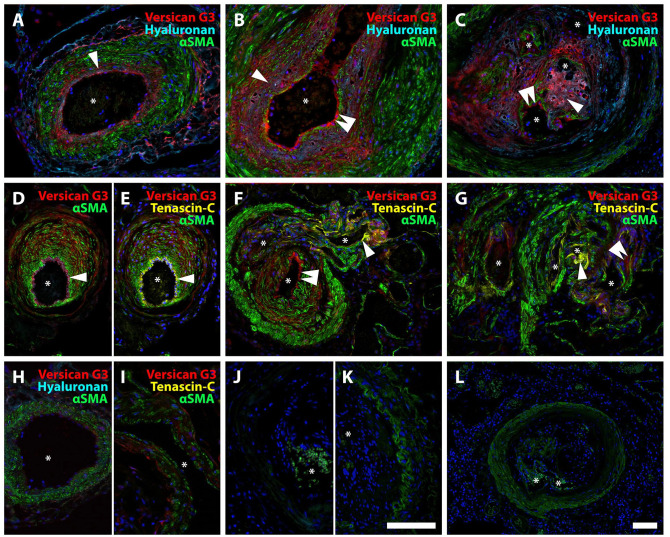
Neointimal accumulation of versican binding partners in IPAH. A-C. Staining pattern for versican G3 and HA in hypertrophied tunica media (A), neointima (B), and plexiform lesion (type 4) (C). Clear co-localization (pink) of versican G3 and HA (single arrowhead); both primarily found in the neointima. Versican G3 was also observed along the endothelial lining (double arrowhead). D-G. Versican G3 (red) and tenascin-C (yellow) with αSMA (green). D, E. Both versican G3 and tenascin-C staining were observed in the endothelium (single arrowhead), although inconsistently for tenascin-C (F, double arrowhead indicating no signal for tenascin-C). Versican G3 was also seen in the tunica media with sparse signal for tenascin-C (D, E, F). In F, a thin-walled vessel is shown exiting a hypertrophied pulmonary artery, whose endothelium is positive for versican G3 but not tenascin-C (double arrowhead), while the thin-walled vessel’s endothelium is tenascin-C positive (single arrowhead). G. Plexiform lesion (type 1) with vascular channels that are either positive for tenascin-C (single arrowhead) or versican G3 (double arrowhead). H. Healthy control, versican G3 and hyaluronan; I. Healthy control, versican G3 and tenascin-C. J. Negative control, versican G3 and HA. K. Negative control, tenascin-C. L. Control, staining for HA on tissue pre-treated with hyaluronidase. Asterisks mark vascular lumen in all images. Scalebars = 100 µm; scalebar in K applicable for A–K.

Staining for the versican G3 domain was strongest in neointimal lesions, but some staining was also seen within hypertrophied tunica media and occasionally in the adventitia of remodeled vessels. In addition, versican G3 frequently colocalized with HA within the neointima and, with the same pattern as DPEAAE, in the endothelium (Fig. 5A to C). Tenascin-C colocalized with versican G3, when present in the neointima. The co-localization was, however, not consistent across all vascular lesions (Fig. 5D to G). Thin-walled vessels originating from either occlusive PAH lesions or plexiform lesions consistently stained positive for tenascin-C, even in the absence of versican G3 and DPEAAE (Fig. 5F to G). Rejected donor lungs displayed little to no vascular accumulation of HA or tenascin-C (Fig. 5H to L).

### Three-Dimensional Epitope and Neoepitope Distribution in Obstructive and Shunt-Type Plexiform Lesions

**Figure 6.**
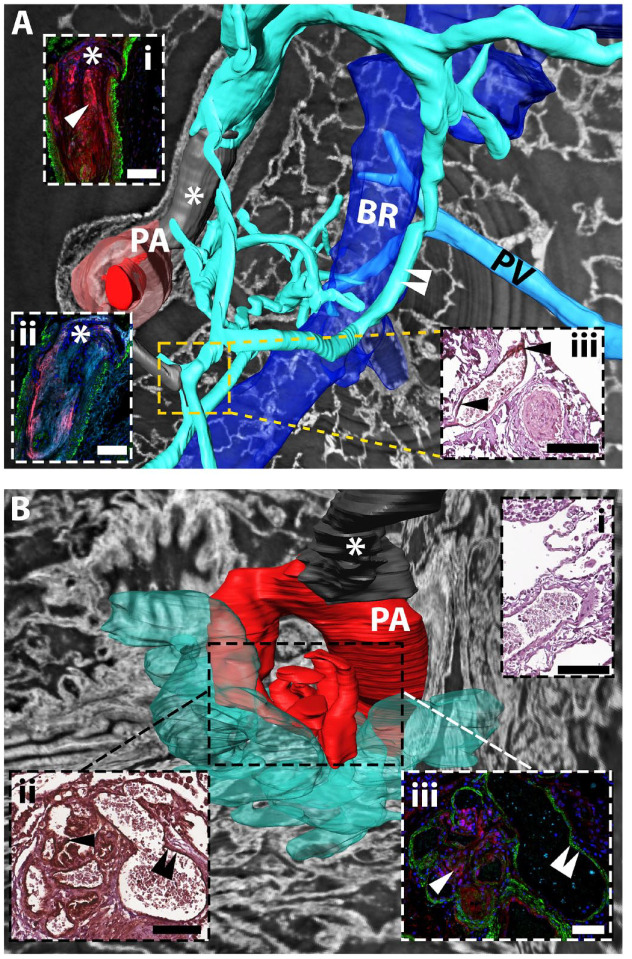
Three-dimensional reconstruction of epitope and neoepitope distribution in obstructive and shunt-type plexiform lesions. A. Type 4 plexiform lesion with bypassing collateral vessels. Dilated thin-walled vessels (turquoise) connect to the type 4 plexiform lesion (gray, asterisk), proximal and distal to the lesion (dark red indicates patent lumen of the pulmonary artery (PA); light red indicates the PA tunica media). The thin-walled vessels twist around the bronchus (BR, blue) and communicate with the peri-bronchial (systemic) circulation (white arrowheads), in addition to directly communicating with the pulmonary vein (PV, seen behind the airway). Inset (i) and (ii) are immunofluorescent staining of the PA occlusive area, inset (iii) immunohistochemistry of the area approximated by the dashed square. Inset (i): versican G3 in red, tenascin-C in turquoise, αSMA in green, arrow indicates co-localization. Inset (ii): DPEAAE in red, hyaluronan in turquoise, αSMA in green: pink = co-localization; (iii) Tenascin-C staining (black arrowheads). Note that the patent thin-walled vessel stains positive for tenascin-C whereas the completely occluded PA does not. B. Type 1 plexiform lesion emerging from a 90° branch of the PA (dark red) proximal to an occlusion (asterisk). Dilated thin-walled vessels (opaque turquoise) emerge from the plexiform lesion and branch out into the parenchyma. Inset (ii) and (iii) are immunohistochemical and immunofluorescent staining, respectively, of the plexiform lesion (dashed square). Inset (i): immunohistochemistry, negative control for immunohistochemical staining for tenascin-C. Inset (ii): positive staining for tenascin-C, in the central part of the lesion (single black arrowhead) and in the endothelium of the thin-walled vessel (double black arrowhead). Inset (iii): staining for versican G3 (red) in the central part of the plexiform lesion (indicated by single white arrowhead) and lack of signal (double white arrowhead) in the thin-walled vessel that stains positive for tenascin-C in inset (ii). αSMA is shown in green. Scalebars = 100 µm. PA, pulmonary artery; BR, bronchiole; PV, pulmonary vein.

Vascular thickening and occlusion, as well as plexiform lesions, were visualized using SRµCT, highlighting the three-dimensional vasculopathy of IPAH. Representative plexiform lesions were manually reconstructed in three dimensions, creating renderings of the vasculature (Fig. 6A to B). We recently reported, through the use of SRµCT, that plexiform lesions not only exist within pulmonary arteries/arterioles, but also at the intersection between the pulmonary and bronchial circulation.^32,34^ Two groups of morphologically distinct plexiform lesions were observed: 1) shunt-type (types 1&2) and 2) obstructive (types 3&4) plexiform lesions. Shunt-type plexiform lesions display connections to the bronchial circulation of either pulmonary arteries or terminal bronchioles,^32,34^ whereas obstructive lesions have no overt connection with the bronchial circulation. However, thin-walled vessels which likely belong to the bronchial circulation can serve as collaterals that by-pass obstructive lesions.

For example, a nearly complete vascular occlusion with some re-canalization (Fig. 6A, type 4 plexiform lesion)^32^ had connections to both pre- and post-stenotic thin-walled collateral vessels, communicating with the bronchial circulation. The type 4 plexiform lesion (occlusive neointima) stained strongly for versican (inset i, red) and HA (inset ii, turquoise), and stained for both DPEAAE (inset ii, red) and tenascin-C (inset i, turquoise) (Fig. 6A). The endothelium of thin-walled collaterals, communicating with the bronchial circulation, stained for tenascin-C (Fig. 6A, inset iii), but did not stain for versican G3 or DPEAAE (data not shown). A type 1 plexiform lesion^32^ displayed co-localization of versican and tenascin-C in the central part of the lesion (Fig. 6B inset iii). Thin-walled vessels that spread out into the parenchyma from this lesion, possibly originating from the bronchial circulation (vasa vasorum), were distinctly positive for tenascin-C, but not for versican (Fig. 6B, inset ii and iii, double arrow). Versican accumulation was thus limited to the site of plexogenic arteriopathy, although its binding partner tenascin-C was present in both pre- and post-stenotic collateral vessels.

### Increased Circulating Versican G3 in PAH Patient Plasma

**Figure 7.**
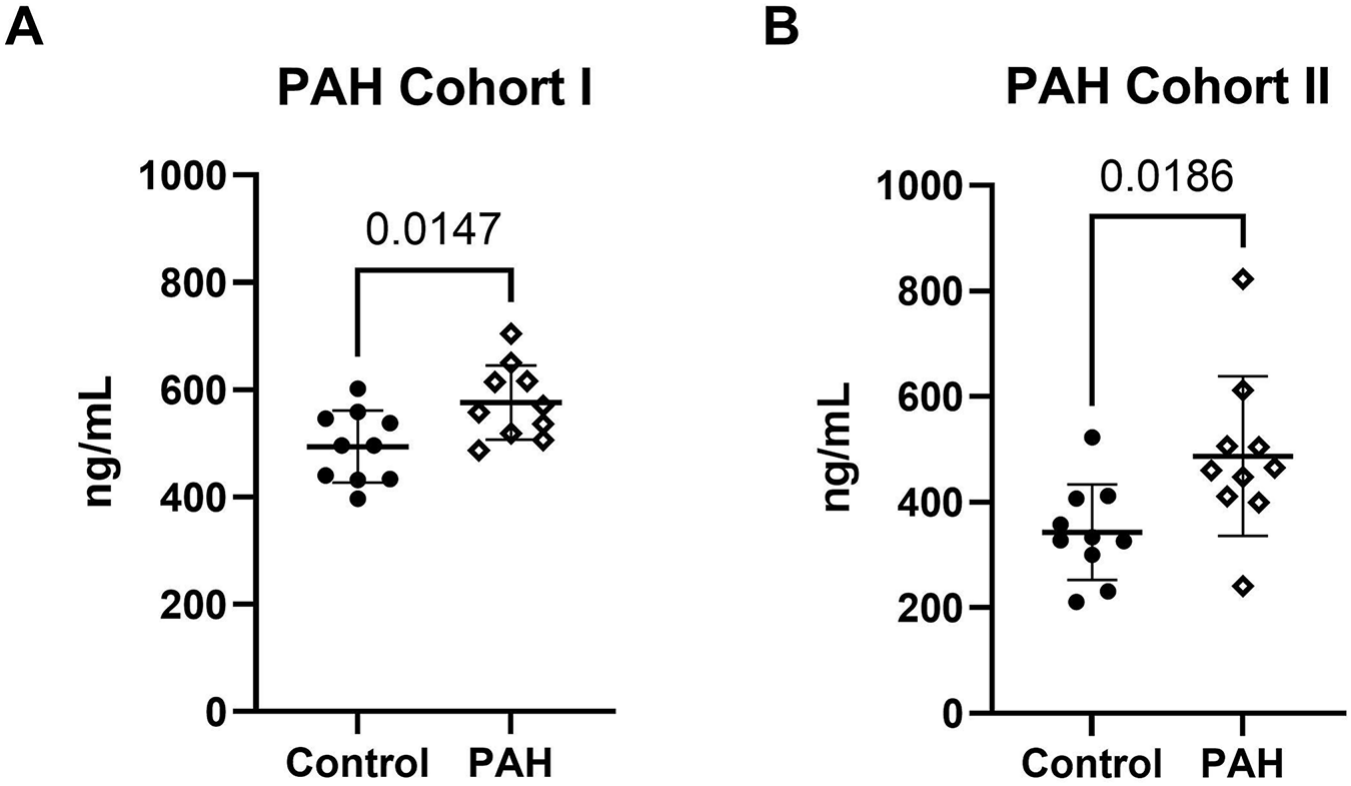
Higher levels of circulating versican G3 in PAH patient plasma. The concentration of versican G3 in control versus PAH plasma was detected in two different patient cohorts using ELISA. Analysis for Patient Cohort I (A) was conducted in triplicates for the experiment shown, while for Patient Cohort II (B), it was done in duplicates. The y-axis represents nanograms per ml (ng/mL). Error bars indicate the mean and standard deviation (SD). An unpaired t-test revealed a significant difference between control (*n* = 10) and patients (*n* = 10) plasma. A p-value < 0.05 was considered significant.

**Appendix Figure 1.**
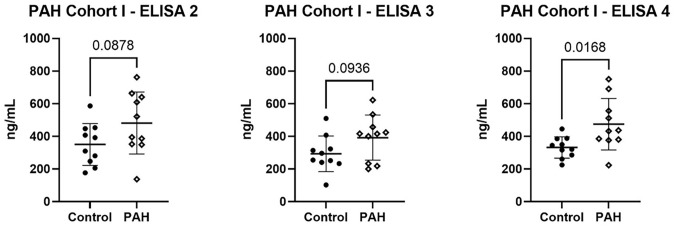
Higher levels of circulating versican G3 in PAH patient plasma—additional technical repeats of PAH Cohort I measurements. Versican G3 in control (*n* = 10) vs PAH Cohort I (A) patient plasma (*n* = 10) detected by ELISA. Data from individual ELISAs are presented, and duplicates in the individual experiments are pooled. The scale on the y-axis is nanograms per milliliter (ng/mL). Error bars represent mean and SD. Two of the technical repeats did not reach statistical significance. PAH, pulmonary arterial hypertension.

N-terminal cleavage of versican V1/V0, represented by the neoepitope DPEAAE, was abundant in the endothelium and neointima of IPAH vasculopathy. Staining for the C-terminal G3 domain of versican was identified in and close to the endothelium, separate from the neoepitope ARRGQF, possibly indicating additional cleavage. Indeed, ADAMTS 1, 4, and 5 are known to cleave versican at multiple sites in addition to the canonical site studied here.^39^ We, therefore, attempted to demonstrate that proteolytic fragments containing the G3 domain of versican were released into the circulation, as was previously shown.^8^ For this, we collected samples from the LCPR, choosing an IPAH/HPAH cohort that resembled the existing tissue cohort (Plasma Cohort I, Table 1) and that had received PAH-specific treatment. We also selected a validation IPAH/HPAH cohort (Plasma Cohort II, Table 1) that had not received PAH-specific treatment. Significantly higher levels of versican G3 was demonstrated in patient samples compared to controls in both patient cohorts (Fig. 7A to B). Notably, we observed variability in significance in repeated measurements in Cohort I. There was however a consistent trend of higher versican levels in IPAH/HPAH patients compared to controls, also for the repeats that did not reach statistical significance (Appendix Fig. 1).

## Discussion

This study aligned with previous reports^6,8^ of versican accumulation in vascular lesions of PAH and illustrated the two- and three-dimensional distribution of versican isoforms, neoepitopes, and their binding partners in the pulmonary vascular tree. ISH confirmed the expression of isoforms containing GAGα and GAGβ within pulmonary vascular lesions. Although GAGβ was seemingly more abundant within remodeled vessels of PAH lungs compared to GAGα, the observation could potentially also result from differential antibody affinity or epitope access. However, our methodologies could not decipher the specific origins of those domains. Since V2 is not expected in lung tissue,^14^ it is likely that full-length V0 is the source for GAGα in remodeled vessels. However, ISH and immunostaining cannot determine whether the GAGβ is within V0 or V1.

The N- and C-terminal ends of versican did not consistently colocalize, suggesting potential motility of the generated fragments following proteolytic fragmentation of versican. In line with this possibility, neoepitopes DPEAAE and ARRGQF were identified within the vascular lesions, affirming ADAMTS-mediated cleavage of V0 and/or V1 in PAH. DPEAAE staining was strongly associated with the vascular endothelium, where versican G3 was also identified in our material. In contrast, endothelial staining of the other epitope created by the same cleavage, ARRGQF, was not observed. The DPEAAE and ARRGQF staining is potentially related to differential G1 and G3 staining, where G1 most often was located in the tunica media. In addition to differential fragment redistribution after E^441-^A^442^ cleavage, further cleavage of the G1-DPEAAE and/or ARRGQF-G3 fragments or interacting partners that could facilitate mobility or alter staining intensity, are also plausible explanations for the patterns observed.

Recent studies highlighted the potential of versikine as a damage-associated molecular pattern that regulates activity of dendritic cells and T-cell infiltration in a context dependent manner.^28,40^ Interestingly, in remodeled pulmonary arteries of PAH patients, the predominant CD45+ cells are lymphocytes, but activated dendritic cells are also present.^41^ A possible impact of versikine on the inflammatory profile of remodeled arteries and on pulmonary arterial endothelium remains to be elucidated.

Ongoing enzymatic cleavage concurs with previous findings^8^ of circulating G3 in plasma from patients with PAH. We confirmed the finding of a higher concentration of versican G3 in IPAH/HPAH patient plasma using ELISA in two separate cohorts from the Swedish LCPR biobank. For plasma samples from Cohort I and II, the aim was to select cohorts that matched the disease severity in the existing tissue cohort to the extent possible, with variance in the PAH-specific treatment. However, hemodynamic and clinical data revealed that plasma Cohort I was markedly healthier with longer 6-minute walk test distances, lower functional class, and lower PVR. It could very well be that the clinical parameters of the plasma cohorts reflect an earlier morphological stage of PAH vascular remodeling, although tissue samples to support this conclusion is lacking. We believe that the milder phenotype seen in plasma Cohort I and II strengthens our conclusion that versican G3 is a possible circulating marker for pulmonary vascular remodeling in PAH. However, during our measurements, we observed intra-assay variability that may have arisen from sample handling, serum-specific matrix effect, and assay variability itself. Additionally, systemically secreted G3 from other organs could affect the result of our analysis. Our interpretation of the relationship between circulating versican G3 and PAH disease severity requires additional studies with larger cohorts.

HA was found in the neointima of PAH lesions, concordant with an elevated concentration in patient plasma as well as in vascular lesions, as previously reported.^17^ In this study, we further demonstrated that HA colocalized with versican G3, possibly indicating the existence of non-cleaved versican core protein, and DPEAAE. Versican-HA complex has been shown to be essential for the proliferation and migration of vascular smooth muscle cells.^21^ As Steffes and colleagues showed, it is likely that dysregulated proliferation of vascular smooth muscle cells is a major factor in neointima formation,^42^ which in turn is predicted to reduce the lumenal diameter and, as such, increase PVR. The relationship between versican and the synthetic phenotype of PASMCs was reported via single-cell transcriptomics, highlighting the modulatory role of versican in this setting.^9^ The osmotic properties of the HA-versican complex are likely to add to this effect but are consistently underestimated in paraffin sections due to tissue dehydration. Interestingly, attempts to inhibit HA synthesis in chronic diseases have been theorized, and to some extent tested, with the use of hymecromone (4-methylumbelliferone), a drug used to treat biliary dyskinesia.^43^ To our knowledge, similar trials in the PAH setting have not yet been undertaken.

Tenascin-C was found in the neointima and also along the endothelium of some, but not all, vascular lesions. Clear positive staining of tenascin-C was, however, also identified along the endothelium of thin-walled vessels associated with intimal and occlusive lesions as well as with plexiform lesions.

In the present study, we have 3-dimensionally reconstructed thin-walled vessels that were associated with vascular occlusions and plexiform lesions. We showed that these thin-walled vessels, to a large extent, were associated with the bronchial microcirculation. These vessels are possibly the result of angiogenesis. In line with this, thin-walled vessels were positive for tenascin-C, a regulator of angiogenesis during wound healing,^44^ but invariably negative for versican G3 or versican neoepitopes. Tenascin-C has previously been reported to be elevated in the peripheral circulation of patients.^18,19^ These results may imply that when elevated tenascin-C concentrations are detected in plasma, a manifest pulmonary vasculopathy with collateral vessels and systemic connections may have developed. Possibly, elevated versican G3 may precede elevated tenascin-C in patient plasma, which could be tested in the future. Animal models, such as the prolonged Sugen5416/hypoxia rat model, shown to produce shunt-type plexiform lesions,^34,45^ could possibly be used to test this along the PAH timeline. The contribution of versican and tenascin-C from other tissues must also be considered.

In conclusion, versican was expressed and localized within vascular lesions of PAH, where it also underwent proteolytic cleavage. Versican and G1/DPEAAE-containing fragments co-localized with HA, which was abundant in all neointimas observed. Versican G3 is strengthened as a potential biomarker for PAH.

## Appendix Information I1

### Immunofluorescence Protocols

Following deparaffinization and rehydration, antigen retrieval was performed using 1X Diva Decloaker (Biocare Medical) at 110°C for 5 minutes in a decloaking chamber (Biocare Medical), followed by blocking with Background Punisher (Biocare Medical) for 10 minutes at RT. One or two primary antibodies (or binding protein), diluted in 1X PBS with 1% bovine serum albumin (Sigma, A7906) were then applied and incubated at 4°C overnight. After washing with PBS buffer, relevant secondary antibodies, or streptavidin, were applied and incubated for 1 hr at RT. Fluorescein isothiocyanate (FITC)-conjugated mouse monoclonal anti smooth muscle α-actin (αSMA) was applied with the secondary antibodies if no anti-mouse secondary was used. Otherwise, it was applied following wash steps with PBS and incubated for 1 hr at RT in the dark. Mounting was performed with ProLong Diamond Antifade Mountant with DAPI (Invitrogen, P36971) or Dako Fluorescence Mounting Medium (Agilent Technologies, S3023); when using the latter, mountant DAPI (Sigma, MBP 0015) was added to the secondary antibody suspension at a concentration of 1:1000. No interaction was observed between the mouse monoclonal anti-smooth muscle actin antibody and secondary anti-mouse Cy3 antibody when applied in separate steps. Blocking with avidin and biotin prior to applying HA-binding protein did not alter the HA-staining pattern (consecutive slides stained and compared) and was therefore not used (not shown). Some slides were treated with chondroitinase ABC (Proteus vulgaris, Sigma-Aldrich, C3667-10UN. 0.2 units/mL in 18 mM Tris-acetate buffer pH 8.0 containing 0.1 % bovine serum albumin) or hyaluronidase (Streptomyces hyaluronolyticus, Sigma H1136-1AMP; 100 units/mL in 125 mM sodium chloride pH 5.2) for 1 hr at 37C prior to blocking. To ensure specificity of the anti-DPEAAE and anti-ARRGQF antibodies, both were incubated with DPEAAE and ARRGQF peptides overnight at 4°C of ratios 1:5 (manufacturer’s instructions) and 1:11, respectively.

## Appendix Information I2

### Immunohistochemistry Protocols

Following deparaffinization and rehydration, antigen retrieval was performed using 1X Diva Decloaker at 110°C for 5 minutes in a decloaking chamber. Endogenous peroxidase activity was quenched with 0.3% H_2_O_2_ in tap water for 30 minutes at RT. Blocking and secondary antibody from the ImmPRESS^®^ Peroxidase detection kit (Vector Laboratories, MP-7402) and substrate from the ImmPACT^®^ DAB Peroxidase (HRP) substrate kit (Vector Laboratories, SK-4105) were used according to the manufacturer’s instructions. Primary antibodies were added following 20 minutes of blocking at RT and incubated at 4°C overnight. Sections were counterstained with Mayer’s Hematoxylin PLUS (HistoLab^®^, 01825) for 3 minutes. Following dehydration, slides were mounted using Pertex (HistoLab^®^, 00840).

**Appendix Table 1.** Concentrations, Specifications, and References for Antibodies Used for Immunofluorescence and Immunohistochemistry.

Antibody	Target	Host	Dilution	Company Reference	Number of Repeats
Anti-versican G1	Versican (hyaluronate-binding region), conserved sequence in N-terminal G1 domain	Mouse (monoclonal 12C5)	1:100	DSHB, 12C5^46^	>5
Anti-versican G3	The exact immunogen used to generate this antibody is proprietary information	Rabbit (monoclonal EPR12277)	1:100	Abcam, ab177480^6^	>5
Anti-DPEEAE	Synthetic peptide within human VCAN aa 400-450, reacts with the C-terminal neoepitope generated by cleavage of the versican core protein by ADAMTS1/4.	Rabbit polyclonal	1:100	Abcam, ab19345 ^47^	>5
Anti-ARRGQF	Versican neoepitope corresponding to the new *N*-terminus generated by ADAMTS versican V1 cleavage at the Glu^441^-Ala^442^ site and V0 cleavage at the Glu^1428^ and Ala^1429^ site ^48^	Rabbit polyclonal	1:100	YZ5769^49^	3
Hyaluronan binding protein, biotin-conjugated	Probe utilized for hyaluronan detection	Recombinant human versican G1 domain (rHABP) expressed in E.coli.	1:100	Amsbio, AMS.HKD-BC41 ^50^	>5
Versican GAGα	Zimmermann et al.^51^	Rabbit	1:100	Zimmermann et al.^51^	3
Versican GAGβ	Zimmermann et al.^51^	Rabbit	1:100	Zimmermann et al.^51^	3
Anti-tenascin-C	Recombinant human Tenascin C	Mouse (monoclonal 4C8MS)	1:50	ThermoFischer, MA5-16086	3
Anti-von Willebrand Factor	Von Willebrand Factor/Factor VIII complex	Rabbit (polyclonal)	1:100	Agilent Dako, A008229-5	3
Alexa Fluor^®^ 488 anti-α smooth muscle actin	The exact immunogen used to generate this antibody is proprietary information	Mouse (monoclonal 1A4)	1:100	Abcam, ab184675	>5
Anti-α smooth muscle actin, FITC-conjugated	The exact immunogen used to generate this antibody is proprietary information	Mouse (monoclonal 1A4)	1:100	Abcam, ab8211	>5
Anti-α smooth muscle actin	The NH2 terminal synthetic decapeptide of α-smooth muscle actin coupled to keyhole limpet hemocyanin (KLH)	Mouse (monoclonal 1A4)	1:200	Millipore Sigma, A2547	>5
Anti-rabbit, Cyanine Cy™3-conjugated	Mouse gamma globulin, heavy and light chain	Goat (polyclonal)	1:200	Jackson ImmunoResearch, 111-165-144	
Alexa Fluor™ 568 anti-mouse	Mouse gamma globulin, heavy and light chain	Goat (polyclonal)	1:200	Abcam, ab175701	
Alexa Fluor™ 568 anti-mouse	Mouse gamma globulin, heavy and light chain	Goat (polyclonal)	1:400	Invitrogen, A11004	
Alexa Fluor™ 488 anti-rabbit	Rabbit gamma globulin, heavy and light chain	Goat (polyclonal)	1:400	Invitrogen, A11008	
Alexa Fluor™ 647 anti-rabbit	Rabbit gamma globulin, heavy and light chain	Goat (polyclonal)	1:200	ThermoFisher Scientific, a21246	
ZyMAX™ Streptavidin-Cy™5	High affinity for biotin	Derived from *Streptomyces avidinii*	1:200	Invitrogen, 43-8316	
